# Risks and Benefits of Fiducial Marker Placement in Tumor Lesions for Robotic Radiosurgery: Technical Outcomes of 357 Implantations

**DOI:** 10.3390/cancers13194838

**Published:** 2021-09-28

**Authors:** Melina Kord, Anne Kluge, Markus Kufeld, Goda Kalinauskaite, Franziska Loebel, Carmen Stromberger, Volker Budach, Bernhard Gebauer, Gueliz Acker, Carolin Senger

**Affiliations:** 1 Department of Radiation Oncology, Charité Universitätsmedizin Berlin, Corporate Member of Freie Universität Berlin, Humboldt-Universität zu Berlin, and Berlin Institute of Health, Augustenburger Platz 1, 13353 Berlin, Germany; melina.kord@charite.de (M.K.); anne.kluge@charite.de (A.K.); goda.kalinauskaite@charite.de (G.K.); carmen.stromberger@charite.de (C.S.); volker.budach@charite.de (V.B.); 2 Charité CyberKnife Center, Augustenburger Platz 1, 13353 Berlin, Germany; markus.kufeld@cyber-knife.net (M.K.); franziska.loebel@charite.de (F.L.); gueliz.acker@charite.de (G.A.); 3 Department of Neurosurgery, Charité Universitätsmedizin Berlin, Corporate Member of Freie Universität Berlin, Humboldt-Universität zu Berlin, and Berlin Institute of Health, Charitéplatz 1, 10117 Berlin, Germany; 4 Department of Radiology, Charité Universitätsmedizin Berlin, Corporate Member of Freie Universität Berlin, Humboldt-Universität zu Berlin, and Berlin Institute of Health, Charitéplatz 1, 10117 Berlin, Germany; bernhard.gebauer@charite.de; 5 Berlin Institute of Health at Charité Universitätsmedizin Berlin, BIH Acadamy, Clinician Scientist Program, Charitéplatz 1, 10117 Berlin, Germany

**Keywords:** CyberKnife, robotic radiosurgery, fiducial marker, tumor tracking, target motion

## Abstract

**Simple Summary:**

Robotic radiosurgery (RRS) allows for the accurate treatment of primary tumors or metastases with high single doses. However, organ motion during or between fractions can lead to imprecise irradiation. We sought to evaluate the risks and advantages of fiducial marker (FM) implantation regarding clinical complications, marker migration, and motion amplitude. Complications were most common in Synchrony^®^-tracked lesions affected by respiratory motion, particularly lung lesions. Pneumothoraces and pulmonary bleeding were the most common complications. An increased complication rate was associated with concomitant biopsy sampling and FM implantation. Most FM migration observed in this study occurred after CT-guided placements and clinical FM insertions. The largest motion amplitudes were observed in hepatic and lower lung lobe lesions. This study highlights the benefits of marker implantation, especially in lesions with a large motion amplitude, including hepatic lesions and lesions of the lower lobe of the lung located >100.0 mm from the spine.

**Abstract:**

Fiducial markers (FM) inserted into tumors increase the precision of irradiation during robotic radiosurgery (RRS). This retrospective study evaluated the clinical complications, marker migration, and motion amplitude of FM implantations by analyzing 288 cancer patients (58% men; 63.1 ± 13.0 years) who underwent 357 FM implantations prior to RRS with CyberKnife, between 2011 and 2019. Complications were classified according to the Society of Interventional Radiology (SIR) guidelines. The radial motion amplitude was calculated for tumors that moved with respiration. A total of 725 gold FM was inserted. SIR-rated complications occurred in 17.9% of all procedures. Most complications (32.0%, 62/194 implantations) were observed in Synchrony^®^-tracked lesions affected by respiratory motion, particularly in pulmonary lesions (46.9% 52/111 implantations). Concurrent biopsy sampling was associated with a higher complication rate (*p* = 0.001). FM migration occurred in 3.6% after CT-guided and clinical FM implantations. The largest motion amplitudes were observed in hepatic (20.5 ± 11.0 mm) and lower lung lobe (15.4 ± 10.5 mm) lesions. This study increases the awareness of the risks of FM placement, especially in thoracic lesions affected by respiratory motion. Considering the maximum motion amplitude, FM placement remains essential in hepatic and lower lung lobe lesions located >100.0 mm from the spine.

## 1. Introduction

For cancer patients, robotic radiosurgery (RRS) is a precise treatment for primary tumors or metastases, delivering high single doses in one or a few radiation sessions [1]. The CyberKnife (CK)-RRS system allows for the delivery of radiation to a hotspot within the target lesion to increase the intensity of the therapeutic effect and enhance the gradient of the dose in the surrounding normal tissues, thus reducing toxicity [2,3,4]. CK-RRS has two unique capabilities: non-isocentric, robotic irradiation, and tracking of moving targets [2]. However, respiratory motion or the involuntary movement of other organs may result in less precise targeting of lesions, adversely affecting nearby organs [5,6,7,8,9]. To overcome this problem, for tumor lesions that are not fixed relative to the spine or skull, one or more fiducial markers (FM) should be implanted within or near the primary tumor (e.g., lung, prostate, and kidney cancer) or metastatic lesion (e.g., lung, adrenal gland, and lymph node lesions) before RRS treatment. FM can be inserted minimally invasively into various extracranial targets to ensure higher precision for moving tumor lesions during one fraction, or in between fractions, while also sparing surrounding tissue because of reduced safety margins [10,11,12,13,14]. For motion compensation, also called “tracking”, the CK uses different techniques. The interaction of precision robotics and an image localization system can compensate for tumor motion via the use of Synchrony^®^ respiratory tracking for lesions that move with respiration, and Fiducial tracking for lesions that do not move with respiration [15,16,17]. Both techniques track the movement of target lesions in real time using FM [18,19].

However, minor and major complications due to marker implantation and FM migration have been reported [12,20,21,22,23,24,25]. FM migration and inter-fiducial geometric changes can lead to inaccuracies or treatment interruptions during RRS [26]. The purpose of this study was to evaluate the risks and benefits of FM implantation prior to Fiducial- or Synchrony-tracked RRS, focusing on technical outcome in terms of complications, marker migration, and motion amplitude.

## 2. Materials and Methods

### 2.1. Study Design

Patients with malignant tumors who underwent FM implantation before CK-RRS at our center between July 2011 and May 2019 were retrospectively identified. The study design was approved by the local ethics review board (EA1/037/20). Clinical data were obtained from patients’ medical records, including age, sex, and site of primary tumor or metastasis. Procedure characteristics, including the FM insertion modality, tracking method, needle size, complications, and marker migration, were also recorded. Adverse effects associated with RRS, such as radiation-induced toxicities, were not investigated. For Synchrony-tracked tumors, we additionally evaluated motion amplitude in relation to the tumor location and the distance to the spine to evaluate the necessity of marker placement.

### 2.2. Implantation Procedure

The FM insertion modality was chosen based on the location of the target lesion. FM implantation modalities include insertion using computed tomography (CT), ultrasound, and endobronchial ultrasound (EBUS) guidance as well as perioperative or clinical FM placements (without imaging guidance). Markers were placed within or in the vicinity of the tumor lesion (usually within 20 mm). As a standard size, a knurled gold marker (1.0 × 3.0 mm or 1.0 × 5.0 mm; Unger Medizintechnik GmbH, Muelheim-Kaerlich, Germany) was used. Other markers were selected as needed. Depending on the interventional radiologist and tumor biopsy requirement, a suitable needle was selected from a range of 17- to 22-gauge for CT-guided procedures. Thinner FM (0.4 × 5.0 mm; Primed Halberstadt Medizintechnik GmbH, Halberstadt, Germany) were implanted in a few centrally located pulmonary lesions via EBUS guidance by an experienced pulmonologist. All FM implanted in the thorax were verified subsequently by chest radiography on the same day as implantation. FM for prostate cancer were typically implanted using ultrasound guidance, while FM for cervical cancers were clinically inserted by a radiation oncologist. For both cancers, the aim was to implant at least three FM, but usually, two pre-spaced gold markers (sterile 18-gauge placement needle with two 1.0 × 3.0 mm FM, spaced by 20.0 mm; FlexiMarc G/T, Riverpoint Medical, Portland, OR, USA) were used to enable rotational tracking. To minimize the risk of FM shift between implantations and the planning CT scan, the procedures were conducted one week apart.

### 2.3. CyberKnife Fiducial Tracking

CyberKnife^®^ VSI Radiosurgery System (Accuray Inc., Sunnyvale, CA, USA) was used for the irradiation. For CK-RRS, two orthogonal live radiographs co-registered with digitally reconstructed radiographs from planning CT images were used to position the patients. Prepositioning was performed using the bony anatomy of the spine, and treatment was administered through the tracking of the implanted FM [27]. Marker detection confidence and marker constellations were checked throughout the procedure. For moving targets, Fiducial tracking was combined with the Synchrony real-time motion synchronization technology. During Synchrony treatments, light-emitting diodes (LEDs) were attached to the patient’s upper body to continuously record respiratory motion. Respiratory motion was synchronized with the internal motion of the FM to determine the position of the FM during different respiratory phases using the radiography system. Thus, the FM position could be predicted at any time based on LED motion, allowing the robotic manipulator of the CK to compensate for movements, using the three-dimensional FM path in near real time.

### 2.4. Assessment of Technical Outcomes

Complications were classified following the guidelines of the Society of Interventional Radiology (SIR) [28], in which minor complications are categorized as class A and B, and major complications are categorized as classes C−F. Class A complications are minimal complications that do not require therapy and are harmless to the patient. Class B complications require either nominal therapy with no harmful consequences for the patient or close observation of the patient, including overnight admission. Class C complications require therapy and hospitalization for less than 48 h, and class D complications require extended therapy with hospitalization for more than 48 h. Class E complications represent permanent impairment, and class F indicates the death of the patient. Additionally, the risk of complications associated with simultaneous biopsy sampling during FM implantation was evaluated.

FM localization was assessed by radiography after marker implantation, on planning CT, or on live stereoscopic radiographs prior to RRS. Patients in whom FM migration had occurred and the marker was not trackable were considered ineligible for CK treatment. Migrated FM were re-inserted in the same session or during a different procedure on a different day. Re-insertion was not performed when an additional intervention was considered impracticable. In these cases, irradiation was performed using existing markers or with spine tracking and by implementing a larger safety margin. All FM implantations that did not require re-implantation and were suitable for CK-RRS were considered technically successful. For Synchrony-tracked lesions, the maximal radial motion amplitude of the FM during RRS was analyzed. The motion amplitude was defined as the range within which 90% of respiratory motion occurred. If a patient received more than one fraction, the motion pattern of all fractions was used. The relationships between motion amplitude and tumor position and distance from the spine were evaluated.

### 2.5. Statistical Analysis

Descriptive parameters are presented as mean ± standard deviation, median, and range. All statistical analyses were performed using IBM SPSS Statistics for Windows/Macintosh, version 25 (IBM, Armonk, NY, USA). Complications related to the method of implantation were assessed via cross-tabulation and the chi-square test. Group comparisons of motion amplitudes were conducted using the Kruskal–Wallis test. *p*-values < 0.05 were considered statistically significant.

## 3. Results

### 3.1. Patient, Lesion, and FM Implantation Characteristics

A total of 357 FM implantation procedures were performed in 288 patients (58% men, 42% women) for 179 primary tumors and 171 metastases (Table 1). In 345 implantation procedures (96.6%), FM were inserted successfully on the first attempt and were suitable for CK therapy. The mean patient age was 63.1 ± 13.0 years (range, 16–87 years). The most common lesions were lung metastases (18.9%), primary gynecologic tumors (18.3%), primary prostate cancer (18.0%), lymph node metastases (14.3%), and primary lung cancer (12.0%). Overall, 725 FM were implanted in 350 lesions, with a mean number of 2.1 ± 1.4 FM per lesion (Table 1). Most FM implantations were performed via CT-guided percutaneous procedures (61.9%), followed by clinically inserted FM (19.3%) and ultrasound-guided FM implantations (16.5%). Other insertion modalities included EBUS (2.0%) and perioperative FM placement (0.3%, Table 2).

Synchrony tracking was used for 191 lesions (226 FM) in 194 implantation procedures, and Fiducial tracking was used for 159 lesions (499 FM) in 163 implantation procedures (Table 3 and Table 4). Synchrony tracking was mostly used for lesions in the thorax (*n* = 137, 71.7%), liver (*n* = 26, 13.6%), and lymph nodes (*n* = 22, 11.5%), while Fiducial tracking was used for lesions in the prostate (*n* = 63, 39.6%), cervix (*n* = 55, 34.6%), and abdominal and pelvic lymph nodes (*n* = 28, 17.6%). A median of one FM was placed (range, 1−3) for Synchrony tracking, and a median of four FM were placed (range, 1−8) for Fiducial tracking.

### 3.2. Marker Complications

**Figure 1:**
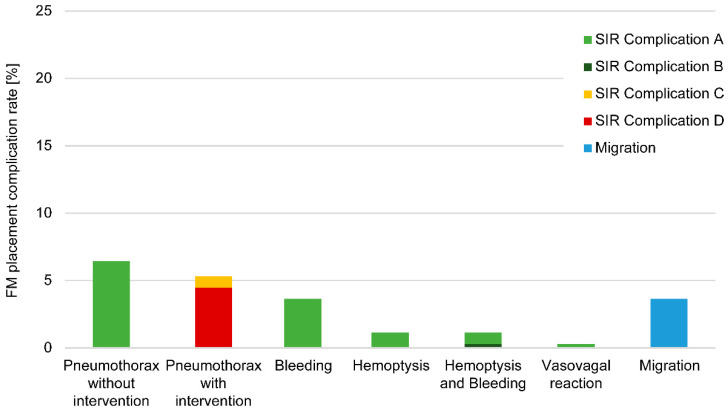
Complications and fiducial marker (FM) migrations. The complication rate of 357 FM placements is shown according to the Society of Interventional Radiology (SIR) classifications.

Overall, 76.8% of the FM placements (274/357) were not associated with complications or migration. The total complication rate was 17.9%, including 45 minor complications (12.6%) and 19 major complications (5.3%). The most common complications included self-limiting pneumothorax (minor complication; 6.4%), pulmonary bleeding and hemoptysis (5.9%), and pneumothorax requiring intervention and >24 h hospitalization (major complication; 5.3%) (Figure 1). Additionally, 3.6% (*n* = 13) of FM migrated from their original location. For six FM insertions (1.7%), the associated complications could not be assessed.

The needle size was evaluated for 204/357 FM implantations, of which 72.1% (*n* = 147) were 18-gauge needles. Among all complications where the needle size was known (*n* = 53), 24.5% were 17-gauge needles, and 75.5% were 18-gauge or smaller needles. The needle size was not associated with complications (chi-square test, *p* = 0.23).

Concomitant tissue sampling data was available for 351 implantations (98.3%), of which 52 underwent concomitant tissue sampling, and 299 did not. Complications were significantly more frequent when concomitant sampling was performed (38.5%) compared to when it was not (16.1%, *p* = 0.001).

#### 3.2.1. *Analysis of Modality-Associated Complications*

The majority of complications were associated with CT-guided FM implantation (*n* = 63, 98.4% of all complications; 28.5% of CT-guided FM implantations; Table 2). Since 59.3% of CT-guided implantations were performed in the thoracic region, most complications were associated with the lungs. Forty-three of the 221 CT-guided FM implantations included class A complications (19.5%), one included a class B complication (0.5%), three included class C complications (1.4%), and 16 included a class D complication (7.2%). Minor complications included self-limiting pneumothorax (*n* = 23, 10.4%), bleeding (*n* = 12, 5.5%), hemoptysis (*n* = 4, 1.8%), or hemoptysis and concurrent bleeding (*n* = 4, 1.8%). All the major complications were pneumothoraces, requiring intervention with drainage (*n* = 19, 8.6%). One case of vasovagal reaction (0.5%) was reported after FM implantation in a pelvic lymph node (Class A).

After ultrasound-guided marker insertion, minimal bleeding was observed in one patient but did not require intervention (class A) (Table 2). There were no complications reported in any other FM implantation modality.

#### 3.2.2. *Locations of Complications*

Most complications occurred in lesions affected by respiratory motion. The complication rate for Synchrony-tracked lesions was 32.0% (62/194 implantations; Table 3). Of these 62 Synchrony-tracked implantations associated with complications, 60 (96.8%) occurred in the thoracic region, including 52 in the lungs (83.9%). Six of eight (75.0%) implantations performed in the middle lobe of the lungs had complications. In the upper lobe of the lung, 27/52 implantations (51.9%) had complications, and in the lower lobe of the lungs, 19/51 implantations (37.3%) had complications. Approximately one-third of implantations in lymph node metastases (31.8%, 7/22 implantations) had complications. The remaining two Synchrony-tracked implantations associated with complications occurred in the abdomen, including one in the kidney (6.3% of 16 kidney implantations) and one in the liver (3.8% of 26 hepatic implantations). No complications occurred with FM implantations in the adrenal gland (Table 3). The complication rate for fiducial-tracked lesions that were not affected by respiratory motion was 1.2% (2/163 implantations, Table 4).

### 3.3. Marker Migration

**Figure 2:**
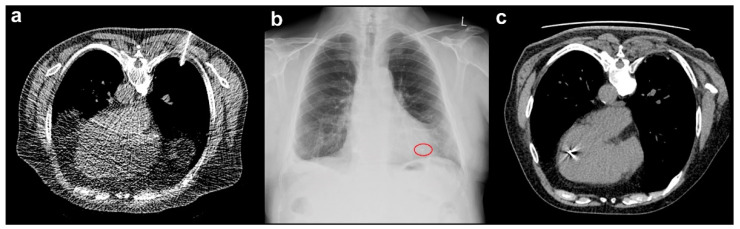
A representative case of marker migration. (**a**) Computed tomography (CT)-guided fiducial marker implantation is used to place a marker into a metastasis in the lower lobe of the right lung. (**b**) Radiography control with the projection of the marker to the heart. (**c**) The marker is shown after migrating to the left ventricle on the day of insertion. There has been no change in the marker position after two years.

FM migration occurred in 13 implantations (3.6%) (Figure 1). The marker was replaced in the same session in six cases, while re-implantation was performed on a different day during the second or third procedure in six cases. The remaining displaced FM was not re-inserted; spine tracking was used instead. In this patient, the FM migrated from a pulmonary metastasis in the right lower lung lobe to the left atrium via the pulmonary vein, ultimately appearing in the left ventricle (Figure 2). After consulting with interventional cardiologists, radiologists, and cardiac surgeons, the marker was not retrieved. At the two-year follow-up visit, the FM remained in the left ventricle, and the patient had no symptoms or complaints after marker migration. FM migration occurred after CT-guided (*n* = 7, 3.2%) and clinical (*n* = 6, 8.7%) implantations (Table 2) and was most common in gynecologic tumors (*n* = 6; Table 4) and pulmonary lesions in the lower lung lobes (*n* = 4; Table 3).

### 3.4. Maximum Radial Motion Amplitude

**Figure 3:**
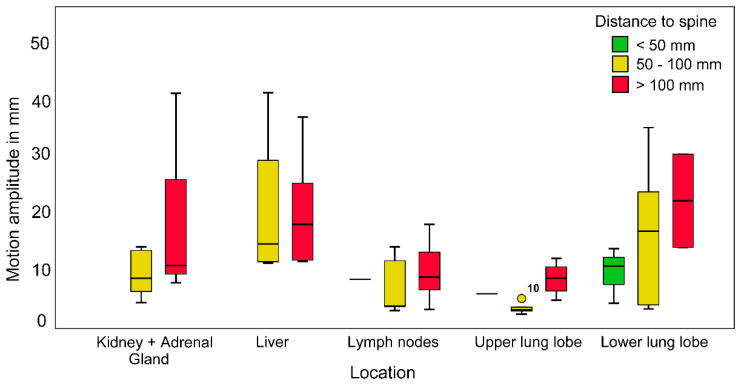
Maximum motion amplitudes of Synchrony-tracked lesions for different tumor locations. The distance from the spine is represented by different colors (green: <50 mm, yellow: 50–100 mm, red: >100 mm). Boxplots show the medians and interquartile ranges, and circles indicate outliers.

Motion pattern data was available for 63 Synchrony-tracked lesions. The maximal motion amplitudes (range, 2.4–40.7 mm) varied based on individual tumor and patient characteristics. The largest motion amplitudes were observed in hepatic lesions (20.5 ± 11.0 mm) and lesions in the lower lobe of the lungs (15.4 ± 10.5 mm). The maximal motion amplitudes differed significantly between tumor locations (Kruskal–Wallis tests, *p* = 0.02). However, there was no significant difference in the distance to the spine (Kruskal–Wallis test, *p* = 0.571, Figure 3). Motion amplitude differences with respect to spine distance can only be observed when individual localizations are considered. Pulmonary lesions in the lower lobe located >100.0 mm from the spine had the largest median motion amplitude (>20.0 mm). Lesions in the lower lung lobe located ≤100.0 mm from the spine, lesions in the kidneys and adrenal glands located >100.0 mm from the spine, and hepatic lesions had median motion amplitudes between 10.0 and 20.0 mm. Tumors in the upper lung lobe and lymph node metastases had median motion amplitudes of <10.0 mm.

## 4. Discussion

This is the second largest study regarding FM implantation. This study specifically focuses on the various anatomical locations of FM implantations. FM increases the precision of radiosurgery, sparing more normal tissue that surrounds moving tumors with a high technical success rate and a relatively low complication rate [10,12,13,21,23]. Despite the clear benefits of FM insertion for radiosurgery, the risks associated with FM placement in patients with cancer cannot be ignored, especially as most candidates for RRS have comorbidities or are receiving palliative treatment. In this series of 357 implantation procedures of 725 markers, successful FM placement on the first attempt with subsequent RRS treatment occurred in 96.4% of patients. The overall complication rate was 17.9%, with 98.4% of complications associated with CT-guided FM placement. Most of the complications were observed in lung lesions, for which all FM implantations were performed with CT guidance. The most severe complication was a pneumothorax requiring intervention and hospitalization (5.3% of all FM implantations), and less severe complications included self-limiting pneumothoraces (6.4%) and pulmonary bleeding and hemoptysis (5.9%).

When the occurrence of complications was compared between lesions affected by respiratory motion (17.4%, 62/357 implantations) and those not affected by respiratory motion (0.6%, 2/357 implantations), Synchrony-tracked lesions in the thoracic region (42.9%, 60/140 implantations), especially in the lungs (46.9%, 52/111 implantations), had higher complication rates. In contrast, abdominal or pelvic FM insertions had extremely low complication rates (3.7%, 2/54 Synchrony-tracked abdominal implantations and 1.3%, 2/157 Fiducial-tracked pelvic implantations). The overall FM migration rate was 3.6%.

The greatest amplitude of motion was noted in lesions in the liver (20.5 ± 11.0 mm) and lower lobe of the lungs (15.4 ± 10.5 mm), with lesions >100 mm from the spine having the largest median motion amplitudes of >20 mm. These results highlight the importance of considering the complication rate, marker migration rate, and expected range of motion according to tumor location when selecting candidates for FM implantation.

### 4.1. Thoracic Complications

Various studies have reported the outcomes of FM implantation [12,13,21,22,23]. The largest FM series reported a technical success rate of 84.0% and an overall complication rate of 3.0% in 616 implantations [12]. The lower complication rate may be due to the fact that only 14.5% of the implantations (89/616) involved pulmonary lesions compared to 31.1% (111/357) in this study. Trumm et al. [21] reported an SIR-defined complication rate of 28.4% in all CT-guided FM placements, which is similar to our results (28.5%). They reported self-limiting and drainage-requiring pneumothoraces (20.0% and 13.3% of cases, respectively) and pulmonary bleeding (33.3%) as complications of FM implantations into pulmonary lesions. Patel et al. [22] also reported self-limiting pneumothoraces (33.0%) and pneumothoraces that required drainage (9.0%). We observed lower complication rates, including the rates of self-limiting pneumothoraces (10.4%), chest tube-requiring pneumothoraces (8.6%), and bleeding (9.1%, including hemoptysis), for CT-guided implantations. Bhagat et al. [23] concluded that FM placement in patients with pulmonary lesions was associated with a high risk of pneumothorax (67.0%). This was attributed to the size, stiffer handling, and lower flexibility of the 18-gauge needle during respiration which can damage the pleura, leading to the development of a pneumothorax. Furthermore, the previous study reported that the duration of the FM implantation procedure may contribute to the risk of pneumothorax, and that small lesions (*p* = 0.03) and lesions not in contact with the pleura (*p* = 0.04) were associated with a higher incidence of pneumothorax. Kothary et al. [13] reported an overall complication rate of 22.3%, which is slightly higher than the rate we observed (17.9%). Most of the complications in Kothary et al.’s study were observed in pulmonary lesions (16.0% involved major complications and 48.0% involved minor complications), with 28/44 patients with pulmonary lesions (63.6%) experiencing complications. The rate of complications associated with pulmonary lesions is much higher than that in our study (46.9%).

Smaller case series have examined endobronchial and endovascular FM placement as alternatives to percutaneous FM placement [25,29,30]. Mongeon et al. [25] compared endovascular FM placement with percutaneous placement and reported no complications associated with endovascular marker insertion. In the percutaneous group, 52.6% of patients had a mild pneumothorax, and 11.4% had a pneumothorax that required chest tube drainage. However, endovascular marker placement is challenging and was recommended for patients ineligible for percutaneous FM implantation. Casutt et al. [29] reported one complication (bronchospasm) among 55 EBUS-guided FM implantation procedures and concluded that the low rate of concurrent transbronchial biopsies may have contributed to the absence of pneumothorax. Majid et al. [30] also inserted FM via EBUS and reported a complication rate of 5.4%. No complications occurred after the endobronchial implantation procedures in this study, though only seven EBUS implantations were conducted. However, significantly thinner FM (0.4 × 5.0 mm vs. 1.0 × 5.0 mm) were often used due to endoscopic limitations, potentially complicating Synchrony tracking.

Bhagat et al. [23] used 18- and 19-gauge needles and found a relationship between the occurrence of pneumothorax and the use of 18-gauge needles (*p* = 0.01). Brook et al. [31] reported major complications for FM placement with simultaneous biopsy sampling alone (*p* = 0.04), although no significant difference was observed for minor complications (*p* = 0.89). Yousefi et al. [32] also identified an association between the development of pneumothorax and concurrent FM implantation and biopsy sampling (*p* = 0.03). Here, no associations between complications and needle size were observed (*p* = 0.23). However, the complication rate was significantly higher when FM implantation was combined with tissue biopsy sampling (*p* = 0.001).

### 4.2. Abdominal and Pelvic Complications

Scher et al. [12] reported very few complications related to FM placement in non-thoracic regions, including two grade 2 complications in hepatic lesions (2/151 procedures) and five grade 1 complications in prostate tumors (5/150 procedures). Saad et al. [33] reported a low complication rate (0.8%; urinary infections and bleeding) in FM implantations in the prostate/prostatic region using the transperineal ultrasound-guided approach, which is similar to our findings. Brook et al. [31] reported SIR-defined complications in 4.3% of implantations in the abdomen or pelvis region using CT- or ultrasound-guided techniques. Kothary et al. [13] reported complication rates of 3.3% in the pancreas, and 2.9% in the liver. The previously reported low complication rates for implantations in the abdominal and pelvic regions are consistent with our findings (1.1% non-thoracic complications, 4/357 implantations).

The complication rate has been reported to be low in hepatic lesions [10,11,24]. Park et al. [10] described only minor complications (11.9%) in their study, which involved ultrasound-guided FM implantation in the liver. Oldrini et al. [11] reported no complications, and Ohta et al. [20] reported only one complication (self-limiting pneumothorax) among 18 implantations (5.6%) using ultrasound- and CT-guided implantation techniques. Choi et al. [24] investigated the efficacy and safety of endoscopic ultrasonography-guided FM implantation in the pancreas and liver and observed mild pancreatitis in 3.1% of patients (1/32). Here, FM placement in the liver was performed via CT guidance, and only one minor (class A) complication was observed. Therefore, both insertion modalities seem equally safe.

### 4.3. Marker Migration

In this study, FM migration occurred in 3.6% of all marker placements and was most frequent in cervical cancer (*n* = 6, 10.2% of cervical implantations) and pulmonary lesions (*n* = 5, 4.5% of lung implantations). Previously reported migration rates are 2.7–19.0% [10,23]. Brook et al. [31] and Park et al. [10] reported similar FM migration rates to this study (4.8% and 2.7%, respectively). However, the FM migration rates reported by Patel et al. [22] and Kothary et al. [13] are higher than that in this study (8.0% and 9.1%, respectively). Bhagat et al. [23] reported a migration rate of 19.0%. Casutt et al. [29] reported a migration rate of 8.0% for EBUS-guided implantations, concluding that the likelihood of migration increased when FM were implanted in a previously irradiated area. Previous irradiation was also a definite cause of FM migration in cervical cancers in our study. Majid et al. [30] suggested that coils, instead of seed markers, may lead to less migration for peripheral pulmonary lesions. Furthermore, the use of paired FM for pulmonary lesions, such as in prostate cancers, may prevent migration. Scher et al. [12] suggested the use of paired FM to reduce the likelihood of marker migration. We used paired FM for prostate and cervical cancer lesions. However, tumor destruction due to concurrent radiochemotherapy and the lack of ultrasound-guided implantation for cervical lesions may contribute to a higher probability of migration. Consequently, FM migration is considered a possible outcome with a low risk.

### 4.4. Motion Amplitude

Several studies have reported similar tumor and marker motilities to those observed in this study. Seppenwoolde et al. [9] reported average motion amplitudes of 9.1 ± 7.3 mm in the lower and middle lobes of the lungs, and 4.1 ± 2.9 mm in the upper lobe of the lungs. We recorded slightly greater maximal motion amplitudes (15.3 ± 10.5 mm in the lower lobe of the lungs, and 6.6 ± 3.2 mm in the upper lobe of the lungs). However, here, lower lobe lesions moved substantially compared to upper lobe lesions, which is consistent with the results of previous reports [9,34]. Additionally, Seppenwoolde et al. [9] found that lower and middle lobe lesions moved an average distance of 1.3 mm when they were attached to bony structures, and 12.5 mm when they were not attached to bony structures. This effect was less pronounced in upper lobe lesions, as lesions attached to bony structures moved an average distance of 3.6 mm, and those not attached to bony structures moved an average distance of 4.2 mm. These findings are similar to our results regarding the distance between lesions and the spine. The average motion reduction in lesions closer to the spine was approximately 50% in this study. There was a clear trend between larger motion amplitudes and greater distances from the spine for lung, kidney, adrenal gland, and lymph node lesions, but not for hepatic lesions. The largest motion amplitudes were noted in the liver (20.5 ± 11.0 mm). Similar radial motion amplitudes (20.9 mm; 95th percentile) were noted for hepatic lesions in a previous study [35].

Previous studies have shown that tracking does not provide an advantage over four-dimensional CT scans with internal target volumes for smaller motion amplitudes (<10.0−15.0 mm) [36,37]. Motion amplitude can be estimated while accounting for lesion location and spine distance. This estimation may help weigh the risks of marker implantation against the benefits, especially in pulmonary lesions. Upper lung lobe lesions are irradiated equally well with spine tracking, which eliminates the risk of complications for implantation. However, spine tracking is not possible for lower lung lobe lesions with larger motion amplitudes, rendering the benefit of the use of FM higher than the possible complications.

### 4.5. Alternative Tracking Modalities

As FM insertion is invasive, we have to discuss the possibility of FM-free RRS treatment. In this regard, Xsight^®^ Lung Tracking System (XLTS) is well known and has been investigated in several studies with similar local control rates compared to FM-based studies [38,39]. However, there are major limitations of this tracking system, such as a certain tumor density, required minimum size, and distance to critical organs, which reduce the number of suitable patients, reflected in a successful tracking rate of only up to 66% [39,40]. Due to technological improvements, there are newer attempts to replace the X-ray FM tracking with CT, magnetic resonance imaging (MRI), or ultrasound at the bedside during RRS treatment, so that abdominal organs can also be treated FM-free [41,42,43,44]. For instance, Rosenberg et al. [42] investigated MRI-guided RRS-treatment in a small cohort of 26 patients for liver lesions. Here, patient and organ motion in the medial/lateral direction might have been missed, and nearby organs at risk for high exposure posed as a limiting factor for the required dose escalation for the target lesion [42]. Similarly, ultrasound-guided tracking has its pitfalls in partial incompatibility between ultrasound and CT scans, the limited field of view, and the fact that quality depends on the experience of the physician. In addition, a very specialized set-up is required [43,44]. Thus, both these technologies must be further developed and become more available to be integrated into everyday clinical practice in the future. Regarding the CT-guided tracking, Papalazarou et al. [41] evaluated a CT-scanner on rails in combination with CK-treatment for pancreatic cancer. The main limitations were the lack of real-time transmission of volumetric data and the possible imprecision in treatment due to patient movement. After the necessary technical improvements have been achieved on the basis of the initial findings, the “CT on rail” is to be integrated into the next generation of CK devices, which will hopefully reduce the need for FM in the future. A very recent study from Moningi et al. [45] compared FM-based vs. FM-free RRS using a CT-based tracking for pancreatic cancer directly. The outcomes indicated that there were no significant differences or disadvantages between the FM-based and the FM-free group in terms of overall survival and progression-free survival. Importantly, patients with more locally advanced pancreatic cancer were more frequent in the FM group than in the FM-free group. Thus, this study again highlighted the need for FM in selected cases and concluded that they did not observe any negative effect of FM on the clinical outcomes of these patients. Another innovative approach to track the tumors in the future could be photoacoustic imaging to detect movements of tumor and organs, which is currently in development [46,47].

### 4.6. Limitations

The major limitation of this study is the retrospective study design. However, only 1.7% of FM implantations had missing data regarding procedural outcomes that prevented the evaluation of complications. The lack of needle size data in some cases may have led to an underestimation of the influence of needle size on the incidence of complications in patients who underwent CT-guided implantation. The rate of marker migration may have also been underestimated due to small migrations not detectable in patients with only one FM implantation. Additionally, motion amplitude could not be determined for all Synchrony-tracked lesions because data regarding RRS treatments was not available until 2016. However, this study is one of the largest series of FM placement in patients with cancer and sufficiently analyzes the efficacy and safety of FM.

## 5. Conclusions

FM implantation can be performed successfully using various modalities in different anatomic regions. Pneumothorax and pulmonary bleeding were the most common complications after CT-guided FM implantation in the thorax for lesions with respiratory motion in this study. However, the complications were either self-limiting or well treatable. Considering the maximum motion amplitude and complications, especially after marker insertion into the thoracic region, FM implantation in upper lung lobe lesions and thoracic lymph node metastases should be carefully evaluated by the physician on a case-by-case basis for individual patients. For lesions in the upper lung lobe and thoracic lymph nodes, similarly precise irradiation can be performed using spine tracking. For hepatic lesions and lesions in the lower lung lobe located >100.0 mm from the spine, FM placement has been proven to be essential because of the large median motion amplitude. There were no significant differences between the implantation approaches in terms of needle size and complication rate; however, additional sampling increased the risk of complications. Paired FM could be used to avoid marker migration in pulmonary lesions. In relation to FM-free tracking modalities, our study supports the overall benefit of the FM-based RRS after this risk assessment until the same precision and local control rates can be ensured for all types of lesions using FM-free RRS.

## Figures and Tables

**Table 1:** Patient and lesion characteristics.

Characteristic	Value
Number of patients	288
Age (years)	63.1 ± 13.0 (16.0–87.0)
Women:men ratio	120:168
Number of lesions	350
Primary tumors	179 (51.1)
Gynecological cancer	64 (18.3)
Prostate cancer	63 (18.0)
Lung cancer	42 (12.0)
Renal cell cancer	10 (2.9)
Metastases	171 (48.9)
Lung	66 (18.9)
Lymph node	50 (14.3)
Liver	26 (7.4)
Adrenal gland	8 (2.3)
Bone	8 (2.3)
Kidney	7 (2.0)
Other	6 (1.7)
Number of implantation procedures	357
Number of fiducial markers	725
Markers per lesion	2.1 ± 1.4 (1.0–8.0)

Data are presented as number, number (percentage), or mean ± standard deviation (range).

**Table 2:** Fiducial marker implantation characteristics.

Implantation Modality	Implantations	FM	Patients	Complications	FM Migration
CT-guided	221 (63.1)	262 (36.1)	160 (55.6)	63 (98.4)	7 (53.8)
Clinical	69 (19.3)	222 (30.6)	63 (21.9)	0 (0)	6 (46.2)
Ultrasound	59 (16.5)	230 (31.7)	59 (20.5)	1 (1.6)	0 (0)
EBUS	7 (2.0)	9 (1.2)	5 (1.7)	0 (0)	0 (0)
Perioperative	1 (0.3)	2 (0.3)	1 (0.3)	0 (0)	0 (0)
Total	357	725	288	64	13

Abbreviations: FM, fiducial marker; CT, computed tomography; EBUS, endobronchial ultrasound; Data are presented as number (percentage).

**Table 3:** Fiducial marker localization and complications in lesions that move with respiration using Synchrony tracking.

Localization	Lesions	FM	Implantations	Complications	FM Migration
Thorax	137 (71.7)	162 (71.7)	140 (72.2)	60 (96.8)	6 (100)
Lung, upper lobe	51 (26.7)	60 (26.6)	52 (26.8)	27 (43.6)	1 (16.7)
Lung, lower lobe	49 (25.7)	58 (25.7)	51 (26.3)	19 (30.7)	4 (66.7)
Lung, middle lobe	8 (4.2)	8 (3.5)	8 (4.1)	6 (9.7)	0 (0)
Lymph node	20 (10.5)	24 (10.6)	20 (10.3)	7 (11.3)	0 (0)
Mediastinum	3 (1.6)	5 (2.2)	3 (1.6)	1 (1.6)	0 (0)
Bone *	6 (3.1)	7 (3.1)	6 (3.1)	0 (0)	1 (16.7)
Abdomen	54 (28.3)	64 (28.3)	54 (27.8)	2 (3.2)	0
Liver	26 (13.6)	34 (15.0)	26 (13.4)	1 (1.6)	0 (0)
Kidney	16 (8.4)	17 (7.5)	16 (8.3)	1 (1.6)	0 (0)
Adrenal gland	8 (4.2)	9 (4.0)	8 (4.1)	0 (0)	0 (0)
Other **	4 (2.1)	4 (1.8)	4 (2.1)	0 (0)	0 (0)
Total	191	226	194	62	6

Data are presented as number (percentage). * includes the ribs (*n* = 3), clavicle (*n* = 1), sternum (*n* = 1), and thoracic wall (*n* = 1). ** includes lymph nodes (*n* = 2), the pancreas (*n* = 1), and abdominal wall (*n* = 1).

**Table 4:** Fiducial marker localization and complications in lesions that did not move with respiration using fiducial tracking.

Localization	Lesions	FM	Implantations	Complications	FM Migration
Pelvis	153 (96.2)	488 (97.8)	157 (96.3)	2 (100)	7 (100)
Gynecological	64 (40.3)	222 (44.5)	68 (41.7)	0 (0)	6 (85.7)
Prostatic	63 (39.6)	229 (45.9)	63 (38.7)	1 (50.0)	0 (0)
Lymph node	26 (16.4)	37 (7.4)	26 (16.0)	1 (50.0)	1 (14.3)
Abdomen	2 (1.3)	2 (0.4)	2 (1.2)	0 (0)	0 (0)
Lymph node	2 (1.3)	2 (0.4)	2 (1.2)	0 (0)	0 (0)
Other *	4 (2.5)	9 (1.8)	4 (2.5)	0 (0)	0 (0)
Total	159	499	163	2	7

Data are presented as number (percentage). * includes the bone (*n* = 2), kidney (*n* = 1), and perianal region (*n* = 1).

## Data Availability

The datasets generated and/or analyzed during this study are not publicly available due to the protection of data privacy. However, they are available from the corresponding author upon reasonable request as an anonymous set.
